# (*E*)-1-(3-Formyl­phen­yl)-2-(2-oxidonaphthalen-1-yl)diazen-1-ium

**DOI:** 10.1107/S1600536813024112

**Published:** 2013-09-04

**Authors:** Assia Mili, Ali Benosmane, Mohamed Amine Benaouida, Abdelkader Bouchoul, Salah Eddine Bouaoud

**Affiliations:** a Unité de recherche de Chimie de l’Environnement, et Moléculaire Structurale, Faculté du sciences exactes, Université de Constantine 1, 25000 Constantine, Algeria

## Abstract

In the title zwitterion, C_17_H_12_N_2_O_2_, the dihedral angle between the benzene ring and naphthalene ring system is 11.76 (7)° and an intra­molecular N—H⋯O hydrogen bond exists. In the crystal, molecules are linked *via* pairs of C—H⋯O hydrogen bonds, forming inversion dimers.

## Related literature

For general background to the use of azo compounds as dyes, pigments and advanced materials, see: Lee *et al.* (2004 ▶); Oueslati *et al.* (2004 ▶). For a related structure, see: Xu *et al.* (2010 ▶).
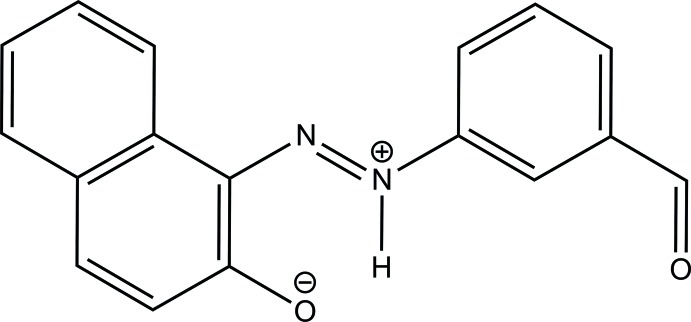


## Experimental

### 

#### Crystal data

C_17_H_12_N_2_O_2_
*M* *_r_* = 276.29
Monoclinic, 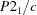

*a* = 5.601 (4) Å
*b* = 7.780 (5) Å
*c* = 29.70 (2) Å
β = 94.624 (16)°
*V* = 1290.0 (15) Å^3^
*Z* = 4
Mo *K*α radiation
μ = 0.10 mm^−1^
*T* = 293 K
0.09 × 0.04 × 0.01 mm

#### Data collection

Nonius KappaCCD diffractometer
16470 measured reflections
3964 independent reflections
2155 reflections with *I* > 2σ(*I*)
*R*_int_ = 0.078

#### Refinement

*R*[*F* ^2^ > 2σ(*F* ^2^)] = 0.057
*wR*(*F* ^2^) = 0.154
*S* = 1.01
3963 reflections
194 parameters
H atoms treated by a mixture of independent and constrained refinement
Δρ_max_ = 0.24 e Å^−3^
Δρ_min_ = −0.23 e Å^−3^

### 

Data collection: *KappaCCD Server Software* (Nonius, 1999 ▶); cell refinement: *DENZO* and *SCALEPACK* (Otwinowski & Minor, 1997 ▶); data reduction: *DENZO* and *SCALEPACK*; program(s) used to solve structure: *SHELXS97* (Sheldrick, 2008 ▶); program(s) used to refine structure: *SHELXL97* (Sheldrick, 2008 ▶); molecular graphics: *ORTEP-3 for Windows* (Farrugia, 2012 ▶); software used to prepare material for publication: *SHELXL97*.

## Supplementary Material

Crystal structure: contains datablock(s) I, global. DOI: 10.1107/S1600536813024112/xu5735sup1.cif

Structure factors: contains datablock(s) I. DOI: 10.1107/S1600536813024112/xu5735Isup2.hkl

Supplementary material file. DOI: 10.1107/S1600536813024112/xu5735Isup3.cml

Additional supplementary materials:  crystallographic information; 3D view; checkCIF report

## Figures and Tables

**Table 1:** Hydrogen-bond geometry (Å, °)

*D*—H⋯*A*	*D*—H	H⋯*A*	*D*⋯*A*	*D*—H⋯*A*
N1—H1⋯O1	0.97 (3)	1.83 (3)	2.577 (3)	133 (3)
C6—H6⋯O1^i^	0.93	2.41	3.327 (4)	168

Symmetry code: (i) 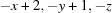.

**Fig. 1.**
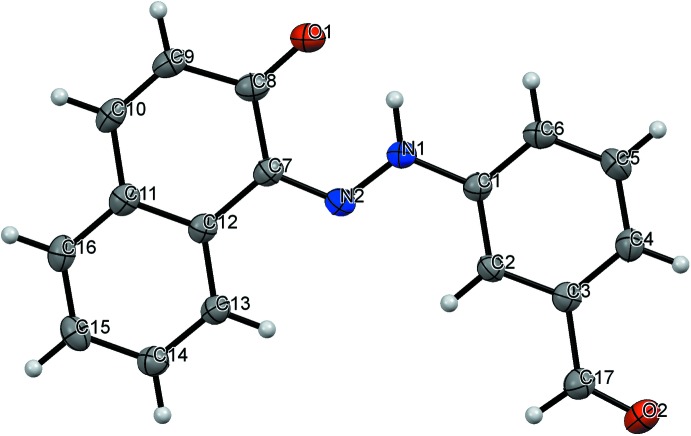
The molecular structure of the title molecule with the atom-numbering scheme. Hydrogen atoms is shown as a small spheres.

## supplementary crystallographic information

### 1. Comment

Azo compounds represent the dominant class of synthetic colourant employed in the textile, printing, agrochemical and pharmaceutical industries (Lee *et al.*, 2004); Oueslati *et al.*, 2004). As a result of the presence of the stable chromophoric azo group (N=N) which is capable of linking different aromatic systems with electron-donating and/or electron-withdrawing groups, dyes can be designed to resist chemical or photochemical degradation processes.

The molecular structure of (I) and the atom-numbering scheme are shown in Figure 1. Two aromatic rings A (C1—C6) and B (C7—C16) show a little deviation from planarity with a dihedral angle of 11.76 °. Intramolecular hydrogen bonds are formed between the phenol hydroxyl groups and the nearest N atom in the 3-aminobenzaldehyde groups [N—H—O = 2.577 (3)], similar to that reported previously (Xu *et al.*, 2010).

### 2. Experimental

Treatment of 3-aminobenzaldehyde (0.02 mol) in 6 ml of 12*M* HCl and NaNO_2_ (0.0214 mol) in 8 ml of H_2_O for 30 min. To the obtained solution, was added dropwise a solution of naphthalen-2-ol, and the resulting brown precipitates were filtrated and washed with water, and dried in a desiccator for several days. Single crystals of I were obtained by slow evaporation from a pentane.

### 3. Refinement

H1 atom was located in a difference Fourier map and refined isotropically. Other H atoms were positioned geometrically with C—H = 0.93 Å and refined in ridding mode with *U*_iso_(H) = 1.2U_eq_(C).

### Crystal data

**Table:**

C_17_H_12_N_2_O_2_	*F*(000) = 576
*M**_r_* = 276.29	*D*_x_ = 1.423 Mg m^−^^3^
Monoclinic, *P*2_1_/*c*	Mo *K*α radiation, λ = 0.71073 Å
Hall symbol: -P 2ybc	Cell parameters from 3964 reflections
*a* = 5.601 (4) Å	θ = 1.3–30.7°
*b* = 7.780 (5) Å	µ = 0.10 mm^−^^1^
*c* = 29.70 (2) Å	*T* = 293 K
β = 94.624 (16)°	Needle, red
*V* = 1290.0 (15) Å^3^	0.09 × 0.04 × 0.01 mm
*Z* = 4	

### Data collection

**Table:**

Nonius KappaCCD diffractometer	2155 reflections with *I* > 2σ(*I*)
Radiation source: fine-focus sealed tube	*R*_int_ = 0.078
Graphite monochromator	θ_max_ = 30.7°, θ_min_ = 1.4°
CCD rotation images, thick slices scans	*h* = −7→7
16470 measured reflections	*k* = −11→11
3964 independent reflections	*l* = −42→42

### Refinement

**Table:**

Refinement on *F*^2^	Primary atom site location: structure-invariant direct methods
Least-squares matrix: full	Secondary atom site location: difference Fourier map
*R*[*F*^2^ > 2σ(*F*^2^)] = 0.057	Hydrogen site location: inferred from neighbouring sites
*wR*(*F*^2^) = 0.154	H atoms treated by a mixture of independent and constrained refinement
*S* = 1.01	*w* = 1/[σ^2^(*F*_o_^2^) + (0.068*P*)^2^ + 0.0107*P*] where *P* = (*F*_o_^2^ + 2*F*_c_^2^)/3
3963 reflections	(Δ/σ)_max_ = 0.002
194 parameters	Δρ_max_ = 0.24 e Å^−^^3^
0 restraints	Δρ_min_ = −0.23 e Å^−^^3^

### Special details

**Table:**

Geometry. All esds (except the esd in the dihedral angle between two l.s. planes) are estimated using the full covariance matrix. The cell esds are taken into account individually in the estimation of esds in distances, angles and torsion angles; correlations between esds in cell parameters are only used when they are defined by crystal symmetry. An approximate (isotropic) treatment of cell esds is used for estimating esds involving l.s. planes.
Refinement. Refinement of F^2^ against ALL reflections. The weighted R-factor wR and goodness of fit S are based on F^2^, conventional R-factors R are based on F, with F set to zero for negative F^2^. The threshold expression of F^2^ > 2sigma(F^2^) is used only for calculating R-factors(gt) etc. and is not relevant to the choice of reflections for refinement. R-factors based on F^2^ are statistically about twice as large as those based on F, and R- factors based on ALL data will be even larger.

### Fractional atomic coordinates and isotropic or equivalent isotropic displacement parameters (Å^2^)

**Table:**

	*x*	*y*	*z*	*U*_iso_*/*U*_eq_	
O1	1.1128 (2)	0.36260 (19)	0.05566 (4)	0.0354 (4)	
O2	−0.0805 (2)	0.98626 (19)	0.12237 (5)	0.0382 (4)	
N1	0.7771 (3)	0.5614 (2)	0.07936 (5)	0.0262 (4)	
N2	0.8540 (3)	0.53524 (19)	0.12186 (5)	0.0244 (3)	
C1	0.5770 (3)	0.6695 (2)	0.06949 (6)	0.0236 (4)	
C2	0.4368 (3)	0.7287 (2)	0.10308 (6)	0.0233 (4)	
H2	0.4746	0.6980	0.1331	0.028*	
C3	0.2409 (3)	0.8333 (2)	0.09152 (6)	0.0252 (4)	
C4	0.1826 (4)	0.8796 (3)	0.04653 (6)	0.0311 (5)	
H4	0.0514	0.9500	0.0388	0.037*	
C5	0.3226 (4)	0.8194 (3)	0.01347 (7)	0.0339 (5)	
H5	0.2836	0.8488	−0.0166	0.041*	
C6	0.5206 (4)	0.7156 (3)	0.02464 (6)	0.0307 (5)	
H6	0.6147	0.6772	0.0023	0.037*	
C7	1.0448 (3)	0.4360 (2)	0.13145 (6)	0.0244 (4)	
C8	1.1796 (3)	0.3516 (2)	0.09708 (6)	0.0275 (4)	
C9	1.3961 (3)	0.2599 (2)	0.11294 (7)	0.0299 (4)	
H9	1.4850	0.2050	0.0921	0.036*	
C10	1.4711 (3)	0.2523 (2)	0.15691 (7)	0.0289 (4)	
H10	1.6132	0.1947	0.1654	0.035*	
C11	1.3401 (3)	0.3301 (2)	0.19198 (6)	0.0260 (4)	
C12	1.1257 (3)	0.4201 (2)	0.17941 (6)	0.0231 (4)	
C13	0.9988 (3)	0.4950 (2)	0.21333 (6)	0.0262 (4)	
H13	0.8574	0.5543	0.2054	0.031*	
C14	1.0815 (3)	0.4817 (2)	0.25807 (6)	0.0291 (4)	
H14	0.9957	0.5322	0.2801	0.035*	
C15	1.2930 (4)	0.3929 (2)	0.27070 (7)	0.0306 (5)	
H15	1.3478	0.3842	0.3010	0.037*	
C16	1.4203 (3)	0.3179 (2)	0.23791 (6)	0.0289 (4)	
H16	1.5610	0.2586	0.2464	0.035*	
C17	0.0939 (4)	0.8931 (2)	0.12780 (7)	0.0299 (4)	
H17	0.1386	0.8564	0.1571	0.036*	
H1	0.862 (5)	0.511 (4)	0.0554 (10)	0.079 (9)*	

### Atomic displacement parameters (Å^2^)

**Table:**

	*U*^11^	*U*^22^	*U*^33^	*U*^12^	*U*^13^	*U*^23^
O1	0.0355 (8)	0.0430 (8)	0.0283 (8)	0.0046 (7)	0.0063 (6)	−0.0055 (6)
O2	0.0316 (8)	0.0381 (8)	0.0459 (9)	0.0057 (7)	0.0096 (7)	−0.0013 (7)
N1	0.0251 (8)	0.0315 (9)	0.0222 (8)	0.0016 (7)	0.0040 (7)	0.0001 (7)
N2	0.0250 (8)	0.0244 (8)	0.0240 (8)	−0.0036 (6)	0.0036 (6)	0.0014 (6)
C1	0.0221 (9)	0.0239 (9)	0.0250 (9)	−0.0017 (7)	0.0026 (7)	0.0012 (7)
C2	0.0228 (9)	0.0274 (9)	0.0197 (9)	−0.0023 (7)	0.0005 (7)	0.0001 (7)
C3	0.0236 (9)	0.0261 (9)	0.0261 (10)	−0.0037 (8)	0.0020 (7)	−0.0011 (8)
C4	0.0278 (10)	0.0339 (11)	0.0312 (11)	0.0044 (8)	−0.0003 (8)	0.0035 (8)
C5	0.0372 (12)	0.0425 (12)	0.0217 (9)	0.0040 (9)	0.0002 (8)	0.0050 (9)
C6	0.0325 (11)	0.0360 (11)	0.0246 (10)	0.0001 (9)	0.0079 (8)	0.0002 (8)
C7	0.0225 (9)	0.0224 (9)	0.0289 (10)	−0.0012 (7)	0.0048 (8)	0.0007 (7)
C8	0.0281 (10)	0.0258 (10)	0.0295 (10)	−0.0039 (8)	0.0081 (8)	−0.0015 (8)
C9	0.0253 (10)	0.0283 (10)	0.0374 (12)	0.0005 (8)	0.0104 (8)	−0.0037 (8)
C10	0.0210 (10)	0.0240 (9)	0.0422 (12)	0.0018 (8)	0.0055 (8)	0.0026 (8)
C11	0.0223 (9)	0.0221 (9)	0.0339 (10)	−0.0032 (7)	0.0037 (8)	0.0015 (8)
C12	0.0203 (9)	0.0223 (9)	0.0270 (10)	−0.0033 (7)	0.0029 (7)	0.0018 (7)
C13	0.0254 (9)	0.0252 (9)	0.0284 (10)	0.0016 (8)	0.0056 (8)	0.0014 (8)
C14	0.0327 (11)	0.0278 (10)	0.0272 (10)	0.0004 (8)	0.0049 (8)	0.0004 (8)
C15	0.0368 (11)	0.0270 (10)	0.0271 (10)	−0.0039 (8)	−0.0035 (9)	0.0036 (8)
C16	0.0248 (10)	0.0250 (10)	0.0362 (11)	−0.0001 (8)	−0.0021 (8)	0.0044 (8)
C17	0.0293 (10)	0.0297 (10)	0.0310 (11)	0.0003 (8)	0.0040 (8)	−0.0023 (8)

### Geometric parameters (Å, º)

**Table:**

O1—C8	1.260 (2)	C7—C8	1.472 (3)
O2—C17	1.217 (2)	C8—C9	1.453 (3)
N1—N2	1.316 (2)	C9—C10	1.340 (3)
N1—C1	1.413 (2)	C9—H9	0.9300
N1—H1	0.97 (3)	C10—C11	1.454 (3)
N2—C7	1.331 (2)	C10—H10	0.9300
C1—C6	1.391 (3)	C11—C16	1.405 (3)
C1—C2	1.397 (3)	C11—C12	1.414 (3)
C2—C3	1.387 (3)	C12—C13	1.405 (3)
C2—H2	0.9300	C13—C14	1.375 (3)
C3—C4	1.397 (3)	C13—H13	0.9300
C3—C17	1.483 (3)	C14—C15	1.396 (3)
C4—C5	1.387 (3)	C14—H14	0.9300
C4—H4	0.9300	C15—C16	1.382 (3)
C5—C6	1.390 (3)	C15—H15	0.9300
C5—H5	0.9300	C16—H16	0.9300
C6—H6	0.9300	C17—H17	0.9300
C7—C12	1.465 (3)		
			
N2—N1—C1	118.93 (16)	C10—C9—C8	121.69 (18)
N2—N1—H1	119.8 (17)	C10—C9—H9	119.2
C1—N1—H1	121.2 (17)	C8—C9—H9	119.2
N1—N2—C7	119.34 (16)	C9—C10—C11	122.90 (18)
C6—C1—C2	120.10 (17)	C9—C10—H10	118.6
C6—C1—N1	117.88 (17)	C11—C10—H10	118.6
C2—C1—N1	122.02 (17)	C16—C11—C12	119.23 (17)
C3—C2—C1	119.72 (17)	C16—C11—C10	121.81 (18)
C3—C2—H2	120.1	C12—C11—C10	118.96 (18)
C1—C2—H2	120.1	C13—C12—C11	118.94 (17)
C2—C3—C4	120.49 (17)	C13—C12—C7	121.84 (17)
C2—C3—C17	118.60 (17)	C11—C12—C7	119.20 (17)
C4—C3—C17	120.91 (18)	C14—C13—C12	120.71 (18)
C5—C4—C3	119.22 (19)	C14—C13—H13	119.6
C5—C4—H4	120.4	C12—C13—H13	119.6
C3—C4—H4	120.4	C13—C14—C15	120.62 (18)
C4—C5—C6	120.87 (18)	C13—C14—H14	119.7
C4—C5—H5	119.6	C15—C14—H14	119.7
C6—C5—H5	119.6	C16—C15—C14	119.64 (18)
C5—C6—C1	119.59 (18)	C16—C15—H15	120.2
C5—C6—H6	120.2	C14—C15—H15	120.2
C1—C6—H6	120.2	C15—C16—C11	120.86 (18)
N2—C7—C12	115.93 (16)	C15—C16—H16	119.6
N2—C7—C8	123.94 (17)	C11—C16—H16	119.6
C12—C7—C8	120.07 (17)	O2—C17—C3	125.28 (19)
O1—C8—C9	121.63 (17)	O2—C17—H17	117.4
O1—C8—C7	121.23 (18)	C3—C17—H17	117.4
C9—C8—C7	117.11 (17)		

### Hydrogen-bond geometry (Å, º)

**Table:**

*D*—H···*A*	*D*—H	H···*A*	*D*···*A*	*D*—H···*A*
N1—H1···O1	0.97 (3)	1.83 (3)	2.577 (3)	133 (3)
C6—H6···O1^i^	0.93	2.41	3.327 (4)	168

Symmetry code: (i) −*x*+2, −*y*+1, −*z*.
